# Supplementary material for: Treatment with IFB-088 Improves Neuropathy in CMT1A and CMT1B Mice
Source: Mol Neurobiol. 2022 Apr 30;59(7):4159–78. doi: 10.1007/s12035-022-02838-y (PMC9167212; doi:10.1007/s12035-022-02838-y)
Supplement: Supplementary file 1 — Supplementary file1 (PDF 1242 KB) [file 12035_2022_2838_MOESM1_ESM.pdf]

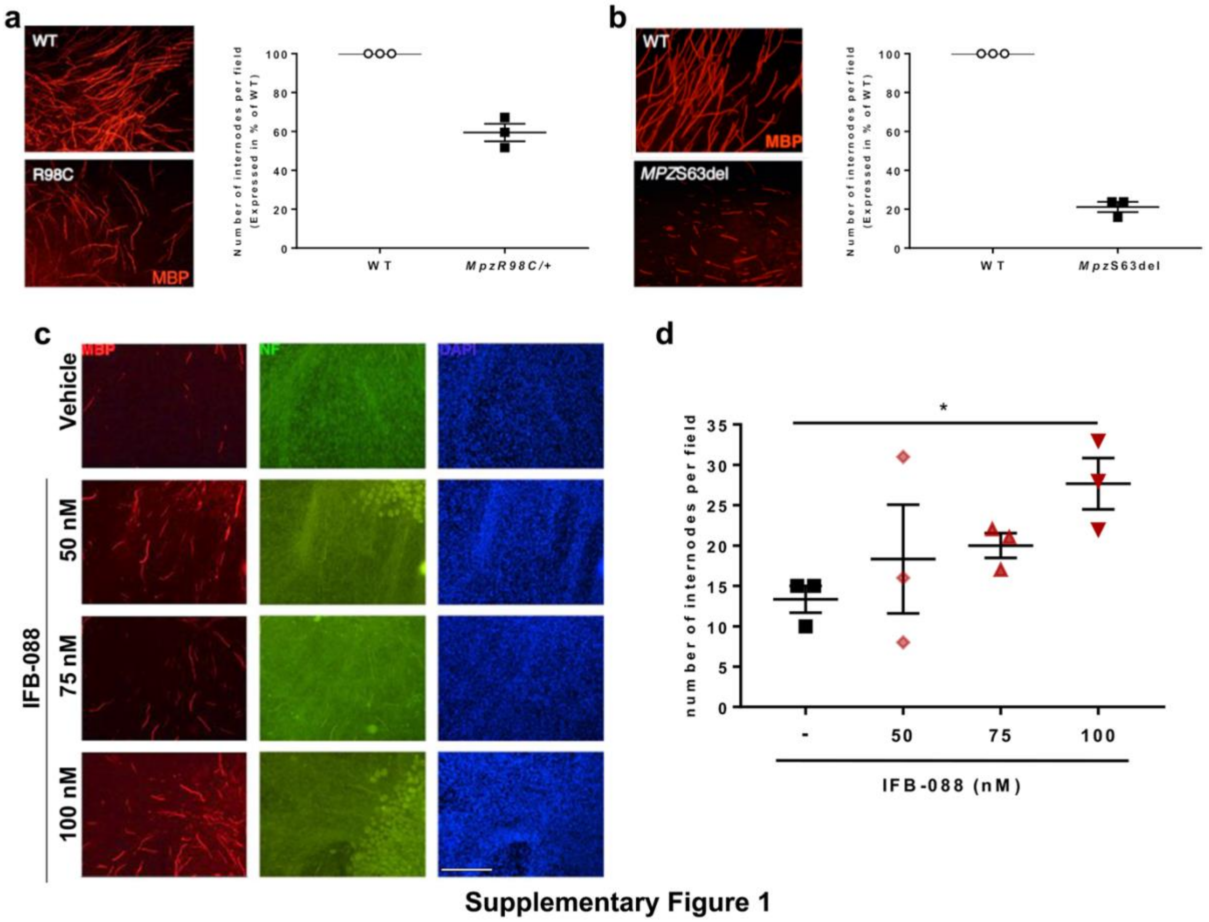

Supplemental Figure 1: IFB-088 improves myelination in DRG explants from *Mpz*S63del mice. Dorsal root ganglia (DRG) were dissected from embryos (E13.5) of *Mpz*S63del mice and *Mpz*R98C/+ mice. The myelination process was induced with ascorbic acid. After 2-week of treatment with vehicle or the indicated concentration of IFB-088, the DRGs were fixed and nuclei visualized by DAPI staining; axons and myelin were visualized by immunostaining with neurofilament antibody (NF) and with myelin basic protein (MBP) antibodies respectively. (**a**) Representative pictures and number of myelinated internodes per field, expressed in % of WT, in WT and *Mpz*R98C/+ DRG explant cultures treated with vehicle for 2 weeks. (**b**) Representative pictures and number of myelinated internodes per field, expressed in % of WT, in WT and *Mpz*S63del DRG explant cultures treated with vehicle for 2 weeks. (**c**) Representative pictures. (**d**) Number of myelinated internodes per field in *Mpz*S63del DRG explant cultures treated with vehicle or the indicated concentration of IFB-088 for 2 weeks. Mean ±SEM. *n*=3 independent experiments. **P*<0.05 One-way ANOVA followed by Friedman's test.

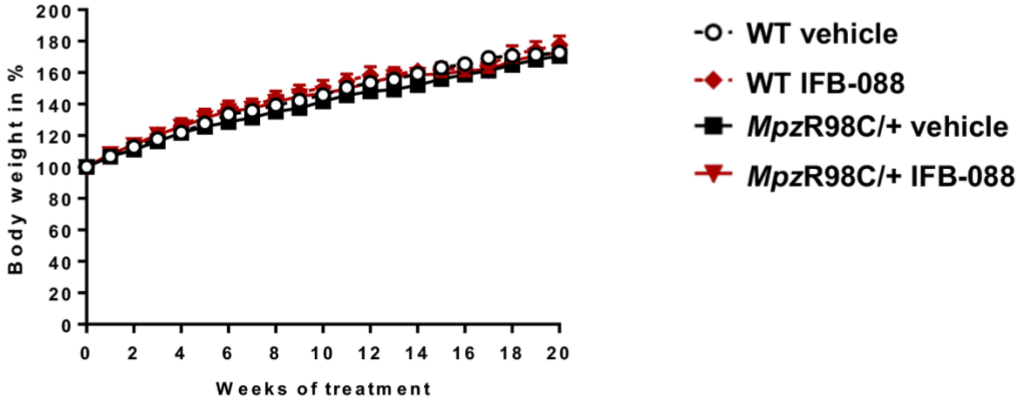

Supplementary Figure 2

Supplemental Figure 2: IFB-088 treatment does not impact WT and *Mpz*R98C/+ mice body weight. Body weight of WT and *Mpz*R98C/+ mice treated with vehicle *b.i.d.* or IFB-088 1 mg/kg *b.i.d.* expressed in % of body weight over 20-week treatment periods (*n*=19-36 mice per condition).

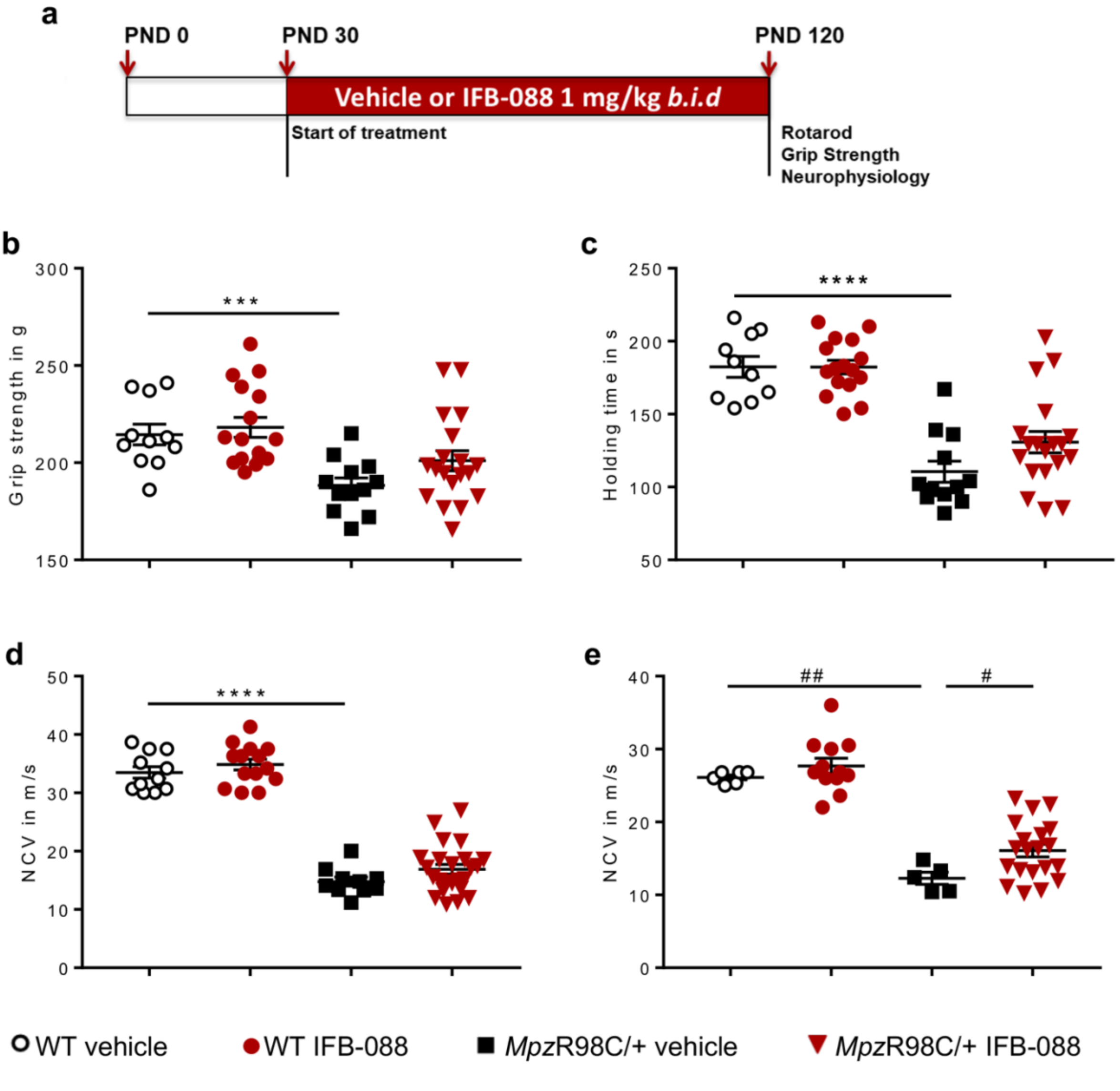

Supplementary Figure 3

Supplemental Figure 3: Impact of 3-month IFB-088 treatment on motor function and nerve conduction velocity in *Mpz*R98C/+ mice. (**a**) Diagram of the treatment strategy. 30-day-old WT and *Mpz*R98C/+ mice were orally administered with vehicle *b.i.d.* or IFB-088 1mg/kg *b.i.d.* for 3 months. (**b**) Four limb grip strength max values average of 10 trials. Data were expressed in grams (g) as mean ±SEM. *n*=11-19 mice per condition. (**c**) Rotarod analysis. Data are expressed in seconds (s) as mean ±SEM. *n*=10-19 mice per condition. (**d**) Motor nerve conduction velocity (MNCV). Data are expressed in meter/second (m/s) as mean ±SEM. *n*=10-24 mice per condition. (**e**) Sensory nerve conduction velocity (SNCV). Data are expressed in meter/second (m/s) as mean ±SEM. *n*=5-20 mice per condition. ***$P<0.001$, ****$P<0.0001$ by Student's T-test; #$P<0.05$, ##$P<0.01$ Mann-Whitney.

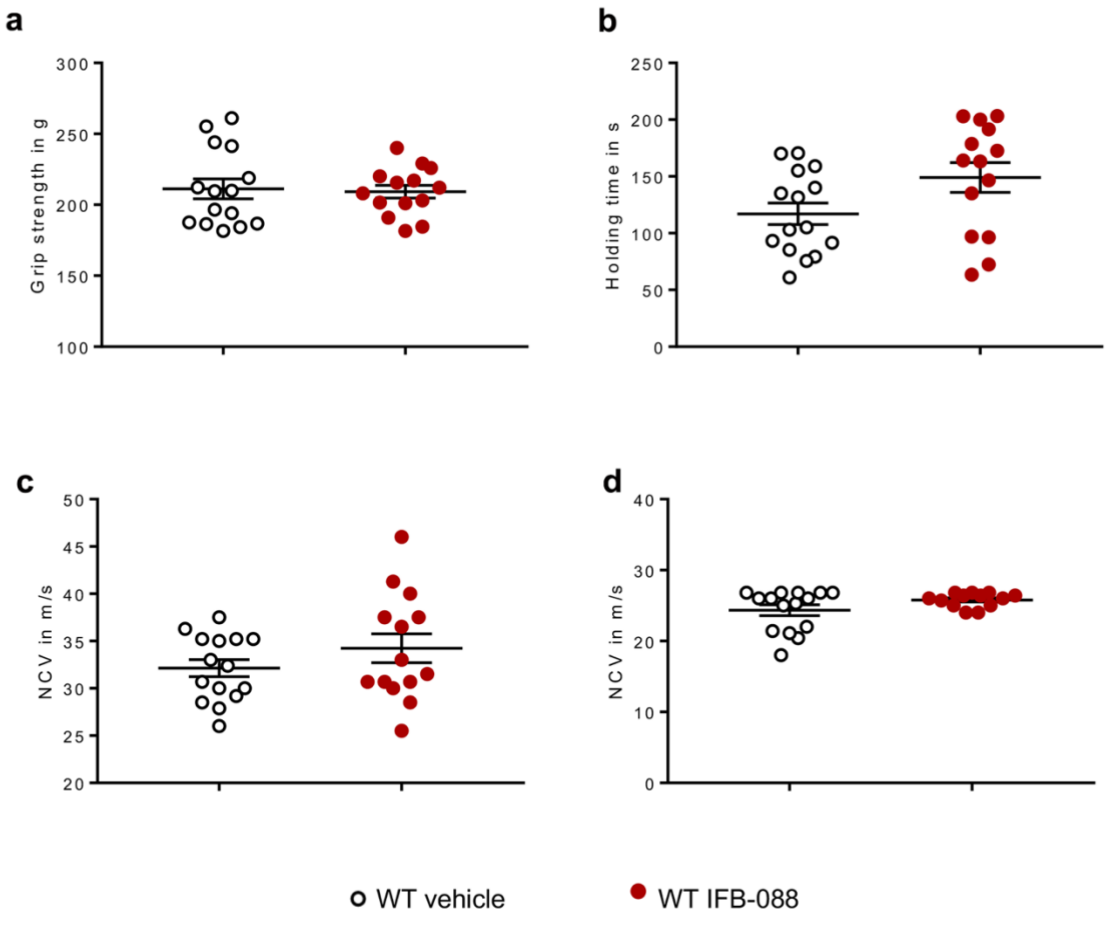

Supplementary Figure 4

Supplemental Figure 4: 5-month IFB-088 treatment does not impact motor function and nerve conduction velocity in WT mice. (a) Four limb grip strength max values average of 10 trials. Data were expressed in grams (g) as mean ±SEM. *n*=14-15 mice per condition. (**b**) Rotarod analysis. Data are expressed in seconds (s) as mean ±SEM. *n*=14-15 mice per condition. (**c**) Motor nerve conduction velocity (MNCV). Data are expressed in meter/second (m/s) as mean ±SEM. *n*=14-15 mice per condition. (**d**) Sensory nerve conduction velocity (SNCV). Data are expressed in meter/second (m/s) as mean ±SEM. *n*=13-14 mice per condition.

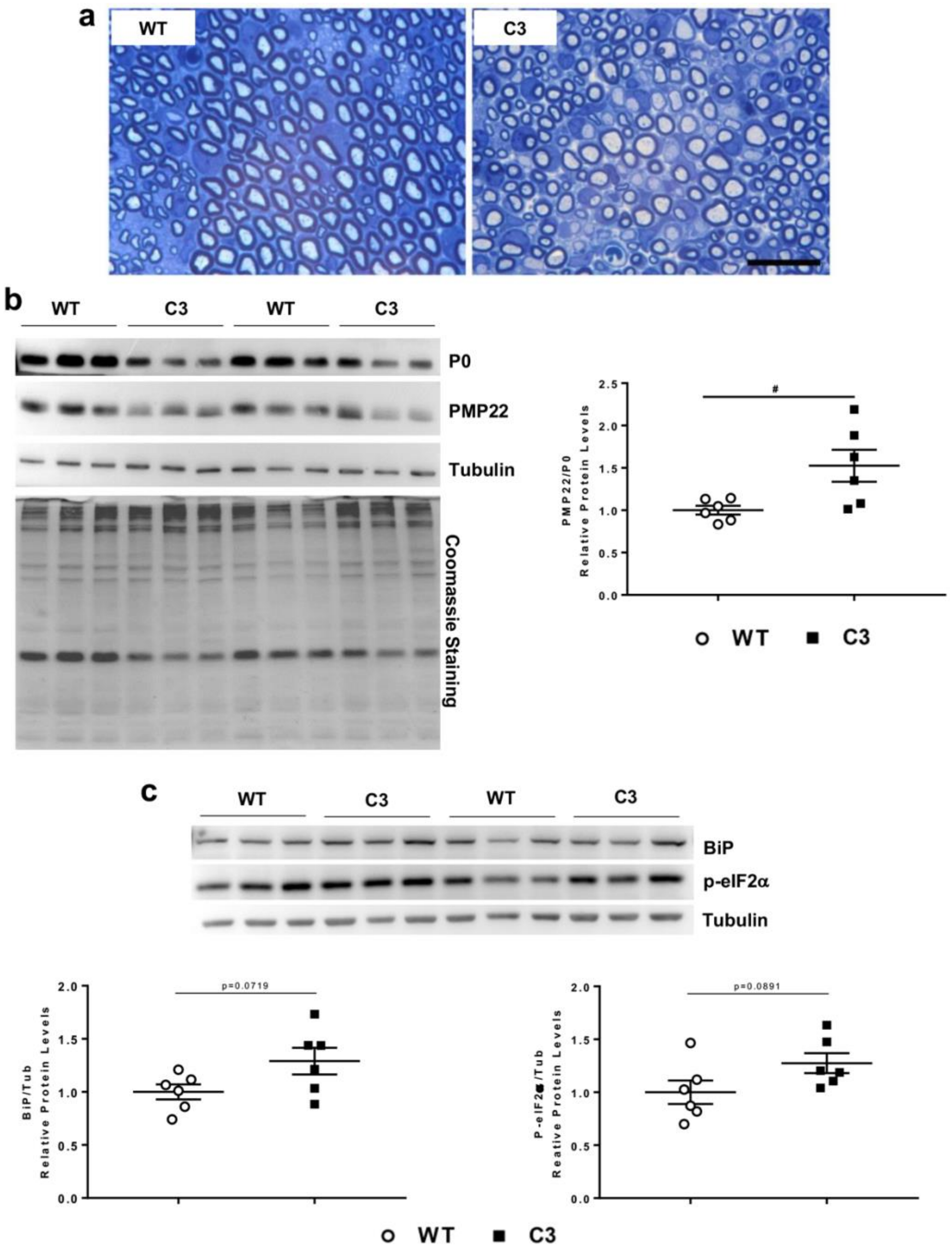

Supplementary Figure 5

Supplemental Figure 5: At PND15, C3-PMP22 mice present a CMT1A-like phenotype with a relative overexpression of PMP22 and expression of ER Stress/UPR markers. (**a**) Toluidine-blue stained transverse semithin-sections from sciatic nerve from 15-day-old WT and C3-PMP22 (C3) mice. Scale bar, 10µm. (**b**) Left: Representative WB for PMP22 and P0 on sciatic nerve protein extracts from 15-day-old WT and C3-PMP22 (C3) mice. Tubulin and Coomassie staining of total proteins were used as loading controls. Right: Quantification of the PMP22/P0 protein ratio relative to total protein. #$P<0.05$ by Mann-Whitney. (**c**) Top: Representative WB for BiP, P-eIF2α and tubulin on sciatic nerve protein extracts from 15-day old WT and C3-PMP22 (C3) mice. Bottom: quantification of protein level from $n=6$ independent nerves. Tubulin was used as loading control. Student's T-test.

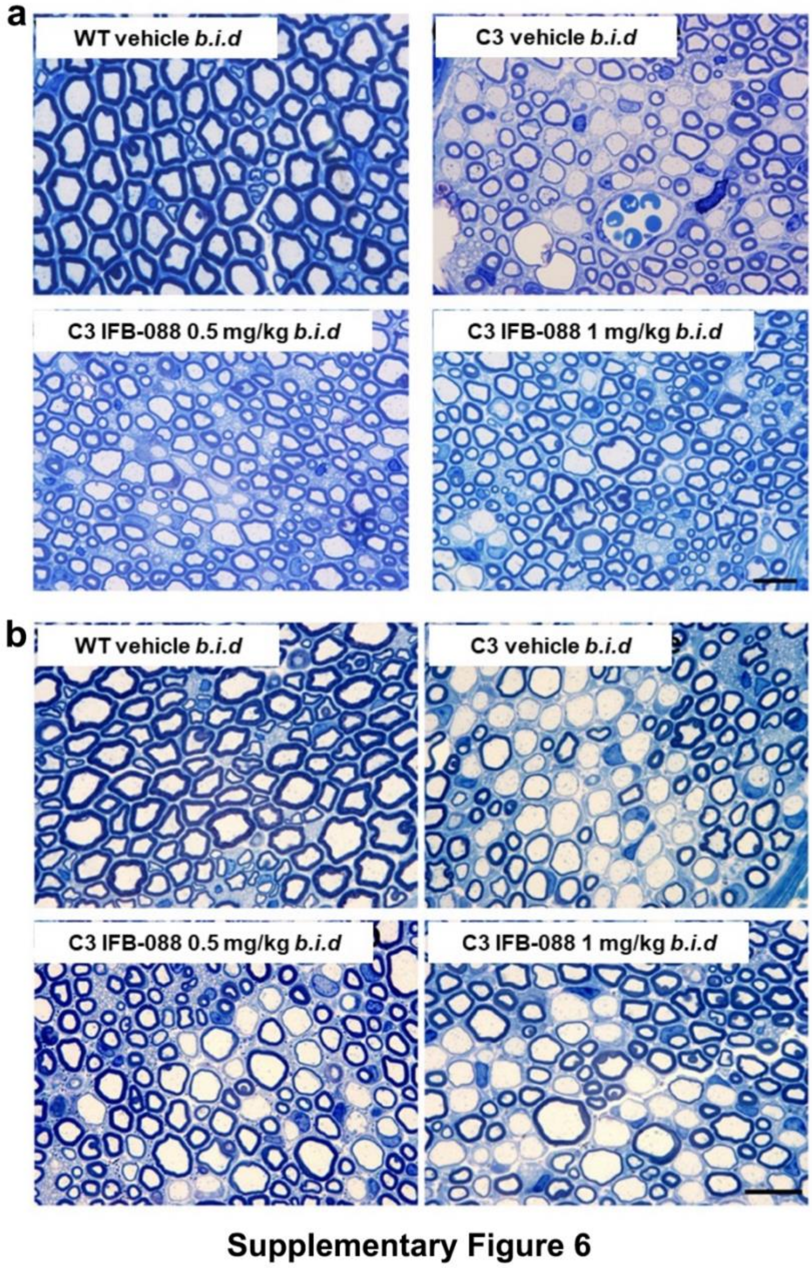

Supplemental Figure 6: Treatment with IFB-088 improves C3-PMP22 mice sciatic nerve morphology. Toluidine blue-stained sciatic nerve sections from (**a**) female and (**b**) male WT mice treated with vehicle *b.i.d.* and C3-PMP22 mice treated with vehicle or IFB-088 at 0.5 or 1mg/kg *b.i.d.* for 12 weeks. Scale bar, 10µm.

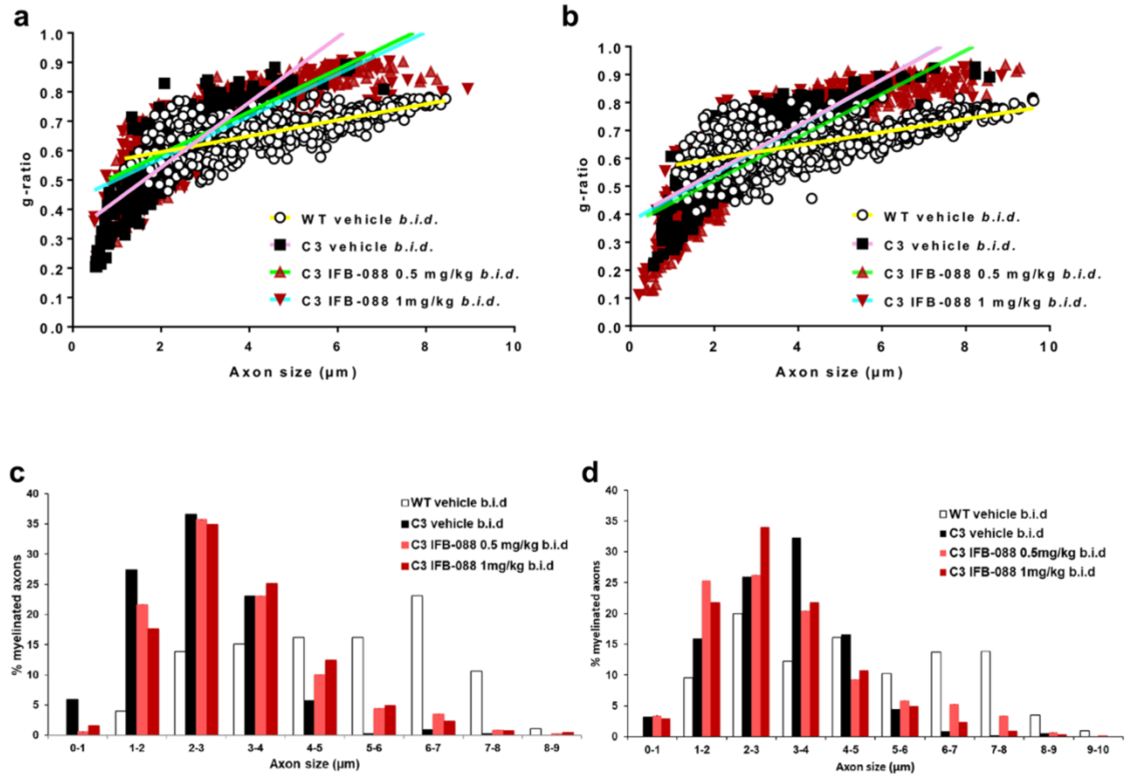

Supplementary Figure 7

Supplemental Figure 7: Treatment with IFB-088 improves morphology of C3-PMP22 quadriceps femoral nerve. Scatter plot of quadriceps femoral nerve g-ratios from WT mice treated with vehicle *b.i.d.* and C3-PMP22 (C3) mice treated with vehicle *b.i.d.* or IFB-088 at 0.5 or 1mg/kg *b.i.d.* (**a**) Females, (**b**) males. Note the "cloud" of axons with diameter lower than 1µm mostly restricted to vehicle treated C3-PMP22 nerves, and the appearance of a considerable number of myelinated axons larger than 5-6µm in treated C3-PMP22 nerves. Percentage of myelinated axons per axons size. (**c**) Females, (**d**) males. *n*=2-4 nerves per condition.

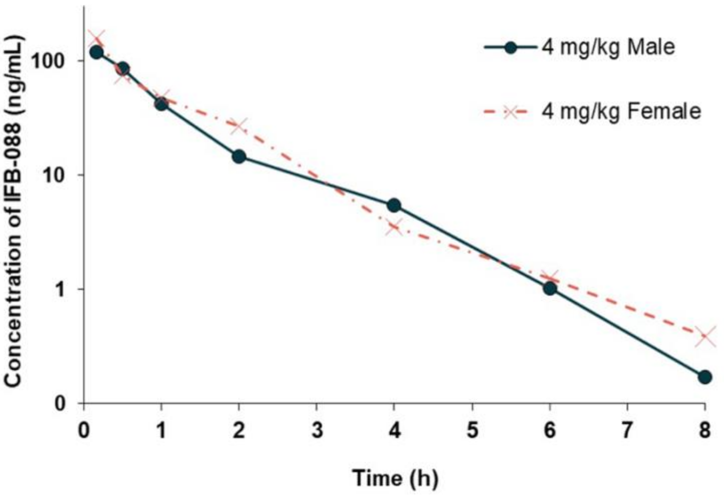

Supplementary Figure 8

Supplemental Figure 8: IFB-088 pharmacokinetic profile. Plasma IFB-088 concentration in C57BL6/J males and females after a single administration at 4 mg/kg. Data are expressed in ng/mL as a mean.
